# Fecal Carriage and Molecular Characterization of Carbapenemase-Producing *Enterobacterales* in the Pediatric Population in Qatar

**DOI:** 10.1128/Spectrum.01122-21

**Published:** 2021-11-10

**Authors:** Andrés Pérez-López, Sathyavathi Sundararaju, Kin Ming Tsui, Hassan Al-Mana, Mohammad Rubayet Hasan, Mohammed Suleiman, Eman Al Maslamani, Omar Imam, Diane Roscoe, Patrick Tang

**Affiliations:** a Division of Microbiology, Sidra Medicine, Doha, Qatar; b Weill Cornell Medical College in Qatar, Doha, Qatar; c Division of Infectious Diseases, Faculty of Medicine, University of British Columbia, Vancouver, Canada; d Biomedical Research Centre, Qatar University, Doha, Qatar; e Division of Pediatric Infectious Diseases, Sidra Medicine, Doha, Qatar; Children’s Hospital Los Angeles, University of Southern California

**Keywords:** *Escherichia coli*, *Klebsiella pneumoniae*, NDM, OXA-48, carbapenemase, carriage, children

## Abstract

Whole-genome sequencing was used to characterize carbapenemase-producing *Enterobacterales* (CPE) strains recovered from rectal screening swab samples obtained from children at a tertiary-care pediatric hospital in Qatar during a 3-year period. A total of 72 CPE isolates recovered from 61 fecal carriers were characterized. Escherichia coli (47 isolates [65.3%]) and Klebsiella pneumoniae (22 isolates [30.6%]) were the most common species identified. High levels of genetic diversity were observed for both species. These 72 isolates produced 78 carbapenemases, characterized as either NDM-type (41 enzymes [52.6%]) or OXA-48-type (37 enzymes [47.4%]). NDM-5 (24 enzymes [30.8%]), NDM-1 (15 enzymes [19.2%]), and OXA-181 (15 enzymes [19.2%]) were the most common variants detected within each type. Twenty-three NDM producers exhibited difficult-to-treat resistance, compared with only 2 of the OXA-48 producers. Multiple comorbidities were identified in 88.5% of the patients, whereas recent travel history to countries in which CPE are endemic was documented for 57.4% of the patients. All 9 *bla*_OXA-48_-type-gene-containing E. coli sequence type 38 (ST38) strains were isolated from patients without international travel history. The mean quarterly incidence of fecal carriage decreased more than 6-fold after the implementation of coronavirus disease 2019 (COVID-19)-related international travel restrictions in Qatar in mid-March 2020. Our data suggest that NDM-type and OXA-48-type carbapenemases expressed by a large diversity of E. coli and K. pneumoniae genotypes are largely dominant in the pediatric population of Qatar. Although our data indicate successful local expansion of E. coli ST38 strains harboring *bla*_OXA-244_ genes, at least within health care settings, *bla*_OXA-48-_type and *bla*_NDM-_type genes appear to have been mainly introduced sporadically by asymptomatic carriers who visited or received health care in some nearby countries in which the genes are endemic.

**IMPORTANCE** To the best of our knowledge, this is the first study addressing the molecular characteristics of CPE in a pediatric population in Qatar using whole-genome sequencing. Since several countries in the Arabian Peninsula share relatively similar demographic patterns and international links, it is plausible that the molecular characteristics of CPE in children, at least in the middle and eastern parts of the region, are similar to those observed in our study.

## INTRODUCTION

The incidence of carbapenemase-producing *Enterobacterales* (CPE) is growing at an alarming pace in many regions of the world, including the Middle East (1, 2), jeopardizing the effectiveness of carbapenems, the last resort to treat infections caused by multidrug-resistant *Enterobacterales* strains, particularly those producing extended-spectrum β-lactamases (ESBLs) and AmpC β-lactamases (3–5).

Qatar is located strategically on the northeast coast of the Arabian Peninsula, with extensive international connections and a large diverse expatriate population, including a significant proportion from Indian subcontinent countries where CPE are highly prevalent (6). Furthermore, although residents of Qatar have traditionally sought medical care abroad, especially in North America and Europe, the recent development of a local modern public health care system is increasingly attracting citizens from neighboring countries and those with trading bonds seeking medical care in Qatar. Therefore, Qatar is vulnerable to the importation and subsequent dissemination of organisms carrying different carbapenemase genes from multiple international reservoirs.

To the best of our knowledge, CPE populations in children in the Arabian Peninsula have never been characterized. We used whole-genome sequencing (WGS) technology to study the molecular characteristics of CPE detected through our proactive surveillance program from pediatric patients over a 3-year period. We also aimed to determine the extent to which the phenotypic and genomic antimicrobial resistance data generated by our WGS data analysis could facilitate optimization of antimicrobial stewardship in children with CPE infections in our institution and to assess whether our infection control procedures are effective in preventing the spread of these microorganisms.

## RESULTS

**FIG 1:**
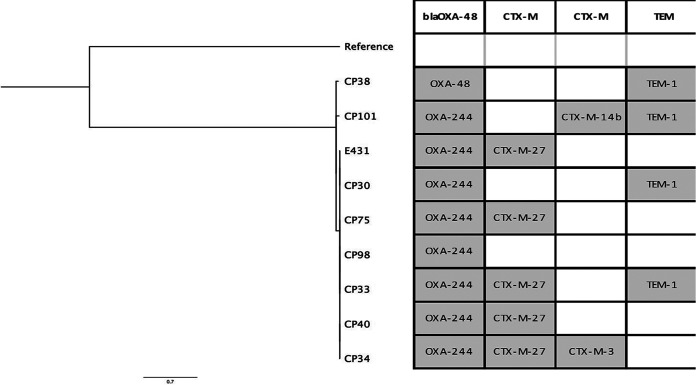
Phylogenetic tree showing genetic relatedness of carbapenemase-producing E. coli ST38 strains. A distance tree showing the genetic relationships among E. coli ST38 samples was reconstructed using RaxML, based on core genome single-nucleotide polymorphisms (SNPs). The tree was rooted with E. coli strain EC958 (accession number GCA_000285655.3). Overlaid on the tree are selected β-lactamase genes predicted for each isolate using ResFinder implemented in ABRicate.

During the study period, 72 unique CPE strains producing 78 carbapenemases were detected and characterized (see Data Set S1 and Tables S1 and S2 in the supplemental material). CPE strains of four species were found, including 47 Escherichia coli strains (65.3%), 22 Klebsiella pneumoniae strains (30.6%), 2 *Citrobacter* sp. strains (2.8%), and 1 Klebsiella oxytoca strain (1.4%). High clonal diversity was observed among the E. coli and K. pneumoniae isolates (see Fig. S1). Twenty-one different sequence types (STs) were detected in E. coli, including ST38 (9 isolates [19.1%]), ST410 (8 isolates [17%]), and ST405 (4 isolates [8.5%]) as the most common clones. Seven of 9 E. coli ST38 isolates were highly similar genetically, but they were different in terms of acquired *bla*_CTX-M_ genes (Fig. 1). In K. pneumoniae, 17 different STs were identified, including ST73 (3 isolates [13.6%]) and ST14 and ST17 (2 isolates [9.1% each]) as the only STs with more than 1 representative.

Among 78 carbapenemases characterized, only NDM-type (41 enzymes [52.6%]) and OXA-48-type (37 enzymes [47.4%]) carbapenemases were detected. The variants identified within each type were NDM-5 (24 enzymes [30.8%]), NDM-1 and OXA-181 (15 enzymes each [19.2%]), OXA-244 (13 enzymes [16.7%]), OXA-48 (7 enzymes [9%]), and NDM-7 and OXA-232 (2 enzymes each [2.6%]). Among E. coli isolates, 26 (55.3%) produced OXA-48-type enzymes and 23 (48.9%) NDM-type enzymes. Among K. pneumoniae isolates, 17 (77.2%) produced NDM-type enzymes and 9 (40.9%) OXA-48-type enzymes. Four (18.2%) K. pneumoniae isolates and 2 (4.2%) E. coli isolates harbored *bla*_NDM_-type and *bla*_OXA-48_-type genes simultaneously (see Data Set S1 and Tables S1 and S2).

Susceptibility patterns for 35 single NDM producers and 31 single OXA-48 producers are shown in Table 1. Twenty-three NDM producers (65.7%) exhibited difficult-to-treat resistance (DTR), compared with 2 OXA-48 producers (6.5%) (odds ratio [OR], 27.8 [95% confidence interval [CI], 5.8 to 136.8]). Among 12 NDM-producing isolates without DTR profiles, 10 were susceptible only to fluoroquinolones, 1 was susceptible only to aztreonam, and 1 was susceptible to aztreonam and fluoroquinolones. Similarly, 31 NDM producers (88.6%) exhibited meropenem MICs of >8 μg/ml, compared with only 1 OXA-48 producer (3.2%) (OR, 232.5 [95% CI, 24.6 to 2,201.7]). Only 2 isolates (2.8%) had colistin MICs that fell within the Clinical and Laboratory Standards Institute (CLSI) non-wild-type category (MICs of ≥4 μg/ml) (see Data Set S1). Nineteen OXA-48 producers for which ceftazidime-avibactam was tested were susceptible to this antibiotic. All of the 70 isolates for which tigecycline was tested exhibited MICs of ≤2 μg/ml. All of the 26 E. coli isolates tested against fosfomycin were susceptible to this antibiotic, according to the breakpoint defined for susceptibility (MIC of ≤64 μg/ml) by the CLSI.

**TABLE 1:** Antimicrobial susceptibility patterns in NDM-type producers and OX48-type producers

Drug	No. (%) of isolates			
	NDM-type producers (35 isolates)		OXA-48-type producers (31 isolates)	
	Susceptible	Nonsusceptible	Susceptible	Nonsusceptible
Amoxicillin-clavulanate	0 (0)	35 (100)	0 (0)	31 (100)
Piperacillin-tazobactam	0 (0)	35 (100)	0 (0)	31 (100)
Ceftriaxone	0 (0)	35 (100)	7 (22.6)	24 (77.4)
Ceftazidime	0 (0)	35 (100)	14 (45.2)	17 (54.8)
Cefepime	0 (0)	35 (100)	9 (29)	22 (71)
Aztreonam	2 (5.7)	33 (94.3)	9 (29)	22 (71)
Ertapenem	0 (0)	35 (100)	1 (3.2)	30 (96.8)
Imipenem	0 (0)	35 (100)	9 (29)	22 (71)
Meropenem	0 (0)	35 (100)	25 (80.6)	6 (19.4)
Gentamicin	18 (51.4)	17 (48.6)	24 (77.4)	7 (22.6)
Amikacin	29 (82.9)	6 (17.1)	27 (87.1)	4 (12.9)
Ciprofloxacin	6 (17.1)	29 (82.9)	16 (51.6)	15 (48.4)
Levofloxacin	11 (31.4)	24 (68.6)	18 (58.1)	13 (41.9)
Trimethoprim-sulfamethoxazole	10 (28.6)	25 (71.4)	10 (32.3)	21 (67.7)
Nitrofurantoin	24 (68.6)	11 (31.4)	28 (90.3)	3 (9.7)

Genes encoding CTX-M-type ESBL were coharbored by 54 isolates (75%), including 39 isolates (54.2%) harboring *bla*_CTX-M-15_ genes, 10 isolates (11.9%) harboring *bla*_CTX-M-14b_ genes, and 5 isolates (6%) harboring *bla*_CTX-M-27_ genes. Cocarriage of *bla*_CTX-M-15_ genes was higher among NDM producers (27 isolates [77.1%]) than among OXA-48 producers (10 isolates [32.3%]) (OR, 7.1 [95% CI, 2.4 to 21.1]); *bla*_CTX-M-27_ genes were detected only in 5 genetically related E. coli ST38 isolates carrying *bla*_OXA-244_ genes (Fig. 1). Genes encoding resistance to antibiotic classes other than β-lactams and quinolone-resistance-determining region (QRDR) mutations are shown in Data Set S1 in the supplemental material. Plasmid-mediated quinolone resistance (PMQR) genes were detected in 41 isolates (56.9%). QRDR mutations were detected in 35 isolates (48.6%). The quadruple mutations of S83L and D87N (*gyrA*), S80I (*parcC*), and S458A (*parE*) were detected in 21 isolates (29.2%), including all E. coli ST410 isolates and all but 1 E. coli ST405 isolates. Nine isolates (12.5%) harbored 16S rRNA methylase (*armA*, *rmtB*, and *rmtC*) genes. All *rmtB* and *rmtC* genes were carried by isolates bearing *bla*_NDM-_type genes, particularly *bla*_NDM-5_ (6/7 isolates [85.7%]). One *bla*_NDM-1_-containing E. coli ST540 isolate with a colistin MIC of 4 μg/ml harbored the *mcr-1* gene (7).

Although a wide range of plasmid replicons was found in 69 isolates (95.8%), including IncFIA (35 isolates [48.6%]), IncFIB(AP0001918) (32 isolates [44.4%]), ColKP3 (16 isolates [22.2%]), IncFIB(K) (15 isolates [20.8%]), and IncX3 (14 isolates [19.4%]), a plasmid location of carbapenemase-encoding genes was identified in 19 isolates (26.4%). All ColKP3 replicons contained *bla*_OXA-48_-type genes (14 *bla*_OXA-181_ and 2 *bla*_OXA-232_), whereas 1 *bla*_NDM-1_ gene was located on an IncA/C2 replicon and 1 *bla*_NDM-5_ gene on an IncFII replicon (see Data Set S1).

The isolates in our collection were carried by 61 patients. Thirty-five (57.4%) had visited or come from a foreign country within 12 months before CPE detection (see Table S1). Sixteen (45.7%) patients visited Indian subcontinent countries (India and Pakistan), 15 (42.9%) Northeast Africa countries (Egypt and Somalia), and 2 (5.7% each) Middle East countries (Kuwait and Iraq) and Turkey. Thirty-two patients (91.4%) with a history of staying in a foreign country had ≥1 underlying medical condition, and 28 (80%) were hospitalized or received medical care in their home or visited countries. Twenty-six patients (42.6%) lacked travel history (see Table S2). Of those, 22 (84.6%) had ≥1 underlying medical condition and 24 (92.3%) had a history of previous hospitalizations and/or residency in long-term-care facilities locally. All E. coli ST38 isolates were recovered from patients without travel history.

**FIG 2:**
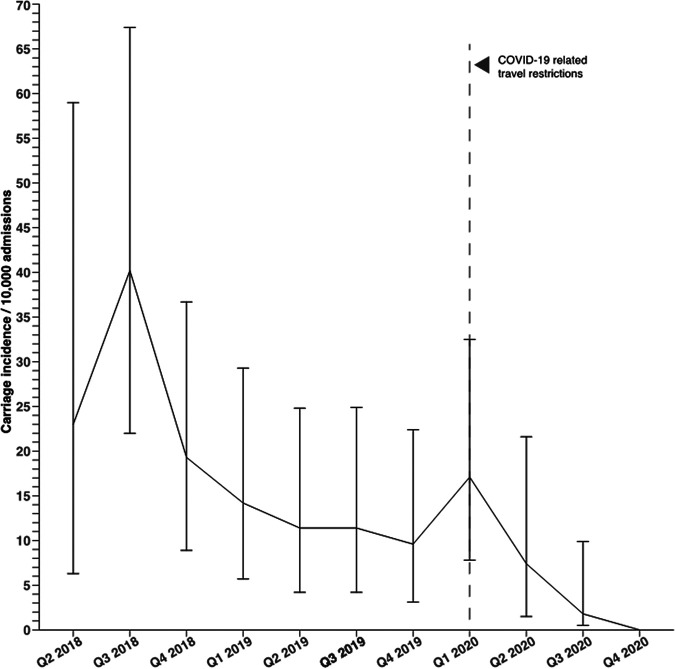
Quarterly incidence of CPE fecal carriage rates per 10,000 hospital admissions from the second quarter (Q2) of 2018 to the end of 2020. Point estimates were calculated for the incidence in each quarter by dividing the number of carriage cases by the number of admissions and standardizing the result to 10,000 admissions. Note the dramatic decrease in CPE carriage incidence after the implementation of COVID-related international travel restrictions in mid-March 2020 in the state of Qatar.

Similarly, whereas the quarterly mean of rectal swab samples processed by our laboratory and pediatric admissions increased slightly, the CPE screening positivity rate decreased almost 7 -fold after the implementation of the coronavirus disease 2019 (COVID-19)-related travel restriction (see Table S3). The mean quarterly carriage incidence decreased from 18.3 cases per 10,000 admissions in the pre-COVID-19-related-travel-restriction period to 3.1 cases per 10,000 admissions in the post-COVID-19-related-travel-restriction period (relative risk [RR], 6.4 [95% CI, 2.3 to 17.7]) (Fig. 2). In our surveillance program, CPEs were not detected in the rectal swab samples from any patients between August and December 2020, except patients who were known CPE carriers. Six fecal carriers were first identified 48 h after hospital admission: Among them, 4 were identified after day 14 of hospitalization in general pediatric units, whereas 2 were detected during their second weekly pediatric intensive care unit (PICU) screening. No neonates admitted to the neonatal intensive care unit (NICU) or other newborn care units were found to be colonized or infected by CPE. Cross-transmission of the same carbapenemase between patients admitted to the same room or hospital unit during the same period did not occur.

## DISCUSSION

Our sequence analysis revealed a limited number of variants of *bla*_OXA-48_-type and *bla*_NDM_-type genes distributed mostly among a great variety of E. coli and K. pneumoniae strains carried by a cohort of children with underlying medical conditions with health care encounters locally or abroad. Our findings concur with those of studies performed in the adult population in Gulf Cooperation Council (GCC) countries (i.e., Bahrain, Kuwait Oman, Qatar, Saudi Arabia, and the United Arab Emirates), pointing out that the molecular epidemiology of CPE in our region is dominated by far by NDM-type and OXA-48-type enzymes produced by multiple clonal linages of both species (8–10). It should be highlighted that KPC-type, VIM-type, and IMP-type carbapenemases, with some notable exceptions, have seldom been reported in our region, suggesting that certain international high-risk clones associated with the spread of specific carbapenemases, such as K. pneumoniae ST258, ST11, and ST307, which are responsible for the dissemination of *bla*_KPC_-type genes elsewhere, have been unsuccessful in spreading across Eastern Arabia (1, 2, 9–12).

In this setting, the remarkable decrease in the mean incidence of CPE carriage registered after the implementation of COVID19-related travel restrictions in mid-March 2020 suggests that asymptomatic sporadic importation from certain regions of the world in which *bla*_OXA-48-_type and *bla*_NDM-_type genes are endemic is playing a predominant role in shaping the molecular epidemiology of CPE in our pediatric population. It should be kept in mind that our infection control practices did not vary and screening rates remained relatively stable throughout the study period (13). However, since all E. coli ST38 strains carrying *bla*_OXA-244_ and *bla*_CTX-M-27_ genes were detected in patients without foreign travel history, the role played by clonal expansion locally was not negligible, suggesting that health care facilities and long-term care institutions in Qatar might be a hidden reservoir for this clone.

In our collection, almost two-thirds of NDM producers exhibited DTR, which contrasted with a small fraction of OXA-48 producers. However, it should be noted that the treatment of the 12 NDM-producing isolates that did not have DTR profiles would be a challenge, given the limited data on the clinical efficacy of monotherapy with fluoroquinolones and aztreonam in NDM infections in children (3, 4). Since 80% of NDM producers coproduced CTX-M-type ESBLs, it could be speculated that susceptibility to aztreonam in those isolates might have been restored by avibactam, making aztreonam-avibactam or ceftazidime-avibactam combined with aztreonam potential rescue therapies for NDM infections in our setting (4, 5, 14). The modest increase in meropenem MICs observed in most OXA-48 producers is noteworthy. In fact, only 1 OXA-48-producing K. pneumoniae strain had a MIC of >8 μg/ml, suggesting that infections caused by these microorganisms, at least in non-critically ill children, could potentially be treated with antibiotic regimens containing prolonged infusion of high-dose meropenem (3, 4). As expected, all OXA-48 producers that were tested against ceftazidime-avibactam were susceptible to this agent, suggesting that this drug could be considered first-line empirical therapy when genes encoding OXA-48 enzymes are detected in clinical isolates (3–5, 14). It is also interesting to note that the proportion of isolates harboring genes encoding 16S rRNA methylases in our collection was smaller than that reported in CPE strains recovered from adults across the region (14), which makes plazomicin a potential therapeutic alternative once pharmacokinetic (PK) and pharmacodynamic (PD) data for this drug in children are available (4). Similarly, although most of our isolates had low MICs for colistin and tigecycline, which are currently used as salvage therapy in combination with other agents, the PK/PD parameters for those antibiotics, including the optimal dosage and therapeutic window in children, have yet to be fully elucidated (3, 4). Our data also suggest that nitrofurantoin and fosfomycin could be viable options to treat uncomplicated lower urinary tract infections caused by carbapenemase-producing E. coli (3). Finally, the presence of multiple QRDR mutations, particularly the double *gyrA* S83L and *parC* 80I mutations, together with PMQR genes among isolates recovered from a group of patients for whom fluoroquinolones are rarely prescribed was particularly striking, suggesting that the selection and spread of certain clones, such as the E. coli high-risk ST405 and ST410 clones, might be facilitated by some fitness advantage conferred by fluoroquinolone resistance (15–17).

Our data suggest that our infection control strategy, based on active surveillance of high-risk patients and implementation of contact precautions upon detection, has been effective in preventing cross-transmission of CPE at the level of colonization pressure experienced by our institution during the study period. In our hospital, asymptomatic carriers and CPE-infected patients are isolated in single *en suite* rooms, cared for by dedicated nursing staff members wearing gloves and gowns during their entire hospital stay, and managed with single-use or decontaminated reusable medical equipment. In addition, rooms are terminally cleaned and decontaminated using hydrogen peroxide vapor following patients’ discharge. Nevertheless, we think that two components of our infection control approach might have played a key role in preventing the hospital spread of CPE. First, our hospital is a newly built facility in which all patients are exclusively placed in private *en suite* rooms, which has been associated with a significant reduction in rates of hospital colonization by some multidrug-resistant organism (MDROs) (18). Second, the overall hand-hygiene compliance rate among hospital staff members increased from 82.7% in 2018 to 94.6% in 2020. Although 6 CPE carriers were first detected 48 h after hospital admission, indicating potential nosocomial acquisition, all of them had just completed treatment courses with broad-spectrum antibiotics before detection (2 with cefepime, 2 with meropenem, 1 with ceftriaxone, and 1 with piperacillin-tazobactam). Therefore, it could be speculated that these isolates might have been acquired before hospital admission and subsequently uncovered by selective pressure exerted by broad-spectrum antibiotics.

Our study has several limitations. First, given that our sample size was small, the actual burden of NDM-type and OXA-48-type enzymes might be overestimated, as genes encoding less prevalent carbapenemase types might also be circulating. Second, since our surveillance protocol did not target otherwise healthy children without contact with health care systems locally or abroad, we were unable to assess the degree to which these genes are circulating among commensal flora in the community. Finally, since our genomic analysis was based on a short-read WGS-based approach, we could not reconstruct the entire plasmids (due to repetitive regions) and determine the precise plasmid location of carbapenemase-encoding genes in the majority of NDM producers (19). Short-read sequencing data also posed a limitation to assessing chromosomal integration of carbapenemase-encoding genes, which has been reported for E. coli ST38 strains harboring *bla*_OXA-48_-type genes in several countries (20).

In summary, *bla*_NDM_-type genes and *bla*_OXA-48_-type genes carried by multiple E. coli and K. pneumoniae clones are largely dominant among CPE in the pediatric population in Qatar. The predominance of E. coli ST38 strains harboring the *bla*_OXA-244_ gene among children with multiple comorbidities and without overseas exposure suggests a clonal spread of this high-risk clone locally. Since several countries in the Arabian Peninsula share relatively similar demographic patterns and international links, it is plausible that the molecular characteristics of CPE in children, at least in the middle and eastern parts of the region, would be similar to those observed in our study. Finally, with CPE established as a major public health threat worldwide, our study emphasizes that WGS is an emerging tool that can be used in clinical practice to optimize antimicrobial stewardship programs targeting these pathogens and to assess the impact of infection control strategies to prevent their spread.

## MATERIALS AND METHODS

### Setting, study design, and patients.

This retrospective observational study was conducted at Sidra Medicine, a 400-bed tertiary-care women’s and children’s hospital with approximately 2,000 pediatric admissions and 75,000 emergency visits per year. The hospital serves as a national referral center for all pediatric medical subspecialties, including oncology, neonatal and pediatric intensive care, and surgical specialties, including cardiac surgery and neurosurgery. Sidra Medicine is a new hospital that started providing outpatient care in January 2016, opened inpatient wards in January 2018, and became fully operational in July 2018, when the emergency department opened.

All nonduplicate CPE strains recovered from surveillance rectal swab samples from children younger than 18 years of age during a 3-year period, between January 2018 and December 2020, were analyzed. In our hospital, risk assessment for CPE colonization is based on a three-question survey at emergency and elective admissions. These questions inquire about the history of colonization with MDROs and the history of having undergone either medical procedures or hospital admission in local or overseas health care facilities in the past 12 months. Patients who answer affirmatively to any of the questions and those who are unsure about the answer to any of the questions are deemed to be at high risk for CPE carriage, and surveillance rectal swab samples are obtained. Patients admitted to the NICU or PICU have routine surveillance swab samples collected on admission and once a week thereafter. Patients hospitalized in other wards for more than 2 weeks are also surveyed weekly. Isolates recovered from the same patient during different hospital admissions were counted as different carriage episodes when the strains were different. Patients’ electronic medical records were reviewed for underlying medical conditions, history of travel abroad, history of overseas medical care, and history of hospitalization or institutionalization in local facilities in the 12 months prior to CPE detection.

### CPE identification and antimicrobial susceptibility testing.

Rectal swab samples were directly inoculated onto CHROMagar mSuperCARBA (CHROMagar Ltd., France) and incubated aerobically in the dark at 35 ± 2°C for 18 to 24 h. Carbapenemase genes in presumptive CPE colonies on chromogenic agar plates were confirmed by the multiplex PCR Xpert Carba-R assay (Cepheid, Sunnyvale, CA, USA). All isolates were identified using matrix-assisted laser desorption ionization–time of flight mass spectrometry (MALDI-TOF MS) (Bruker, Germany) and tested for antimicrobial susceptibility using the BD Phoenix automated susceptibility testing system (Becton, Dickinson, USA). MICs and breakpoints were interpreted according to CLSI 2019 guidelines (21). Isolates with MICs in the intermediate and resistant ranges were grouped in a single category as nonsusceptible. Since CLSI guidelines provide interpretive criteria only for fosfomycin tested against Escherichia coli, the susceptibility to this agent was studied only in this species. In addition, MICs for colistin were determined by broth microdilution (ComASPTM Colistin test panel; Liofilchem, Italy), and isolates were separated into wild-type and non-wild-type groups based on the epidemiological cutoff values provided by the same CLSI guidelines (21). We defined DTR as resistance to all β-lactam and fluoroquinolone antibiotics (22). Isolates that were confirmed to have a carbapenemase gene underwent subsequent WGS analysis. Clinical species harboring chromosomal *bla*_AmpC_-type genes, such as Enterobacter cloacae complex and Citrobacter freundii strains with reduced susceptibility to ertapenem only, were inferred to have overexpression of their AmpC β-lactamase combined with reduced porin expression and were consequently excluded (23).

### WGS and bioinformatic analysis.

Genomic DNA was extracted using the NucliSENS easyMag system (bioMérieux, Marcy-l’Etoile, France). Isolates were sequenced (paired-end reads with a read length of 300 bp) on the Illumina MiSeq platform with MiSeq reagent kit v3. Raw reads were assessed by FastQC (http://www.bioinformatics.babraham.ac.uk/projects/fastqc) and quality trimmed by Trim Galore (http://www.bioinformatics.babraham.ac.uk/projects/trim_galore) to eliminate low-quality sequences. Trimmed reads were assembled using SPAdes v3.9.0 (24). The genome assemblies were assessed using QUAST v5.0.2 (25), and contaminant reads were excluded. STs and the genes encoding antimicrobial resistance were predicted based on the PubMLST typing schemes implemented in mlst (https://github.com/tseemann/mlst) and the ResFinder v3.2 database (26) implemented in ABRicate v0.9.8 (https://github.com/tseemann/abricate). Mutations in the QRDRs were detected using AMRFinderPlus v3.8 (27). The plasmids were determined based on the PlasmidFinder v2.1 database (28). The genetic relatedness among E. coli and K. pneumoniae strains was inferred using Parsnp v1 (29).

### Statistical analysis.

ORs and their 95% CIs were used to assess the strength of association between the type of carbapenemase and antimicrobial resistance patterns. Carriage incidences were calculated with the quarterly number of newly identified carriage episodes and expressed by 10,000 hospital admissions. The state of Qatar implemented international travel restrictions on 15 March 2020 to prevent the spread of COVID-19, which resulted in a 95% reduction in the number of international arrivals in Qatar during the rest of 2020, compared with the same period in 2019 (13). In this context, the RR and its 95% CI were reported to compare the risk of CPE carriage between two periods, i.e., before (second quarter of 2018 to first quarter of 2020) and after (second quarter of 2020 to fourth quarter of 2020) the implementation of COVID-19-related travel restrictions.

### Ethics.

This study was approved by the institutional review board of Sidra Medicine (protocol number 1804022140).

### Data availability.

Raw sequence data are available in GenBank under BioProject accession number PRJNA667192.
