# Supplementary material for: Fecal Carriage and Molecular Characterization of Carbapenemase-Producing Enterobacterales in the Pediatric Population in Qatar
Source: Microbiol Spectr. 2021 Nov 10;9(3):e01122-21. doi: 10.1128/Spectrum.01122-21 (PMC8579929; doi:10.1128/Spectrum.01122-21)
Supplement: SUPPLEMENTAL FILE 2 — Supplemental material. Download SPECTRUM01122-21_Supp_1_seq7.pdf, PDF file, 0.6 MB [file spectrum01122-21_supp_1_seq7.pdf]

Supplementary Table S1. Carbapenemen-producing Enterobacterales isolated from fecal carriers with history of foreign travel and/or medical care overseas.

ID	Age/Gender	Underlying condition	Foreign country visited	Medical care overseas	Species/ST	Enzymes
**CP3**	11 years/F	Duodenal peptic ulcers	India	India	*E. coli*/617	NDM-5
**CP5**	4 months/M	Imperforate anus & Colonostomy	Somalia	Sudan	*K. pneumoniae*/14	NDM-1
**CP8**	18 years/M	CP, GDD & Epilepsy	Pakistan	Pakistan	*E. coli*/354	OXA-181
**CP9**[a]	6 months/F	Down syndrome, CHD & Cardiac surgery	Pakistan	Pakistan	*E. coli*/540	NDM-1
**CP15**	2 years/M	Previously healthy	Egypt	Egypt	*E. coli*/167	NDM-5
**CP16**	1 year/M	CP, GDD, RLRTI	India	India	*C. freundii*/ND	NDM-1
**CP17**	1 year/M	Previously healthy	Egypt	NO	*E. coli*/405	NDM-5
**CP18**[b]	6 years/M	HLH	India	India	*E. coli*/410	NDM-5
**CP19**[b]	6 years/M	HLH	India	India	*K. pneumoniae*/2096	OXA-232
**CP20**	11 months/M	Alexander disease, Hydrocephalus & VPS	Egypt	Egypt	*E. coli*/10	NDM-5
**CP21**	6 years/M	Previously healthy	India	NO	*E. coli*/46	NDM-5
**CP22**	1 month/F	Born and NICU admission in India	India	India	*K. pneumoniae*/337	NDM-1

**CP70**	10 month/M	Prematurity & bladder Exstrophy surgery in Qatar	Egypt	NO	*K. pneumoniae*/441	OXA-181
**CP23**[c]	11 months/M	Down syndrome, CHD & Cardiac surgery	Pakistan	Pakistan	*E. coli*/405	NDM-5
**CP36**	4 years/M	Perforated appendicitis & Intraabdominal abscess	Egypt	NO	*E. coli*/410	NDM-5
**CP25**	1 year/M	Retinoblastoma	Egypt	Egypt	*E. coli*/3541	OXA-244
**E341**[c]	1 year/M	Down syndrome, CHD & Cardiac surgery	Pakistan	Pakistan	*K. pneumoniae*/336	NDM-1
**CP28**	9 years/F	Osteosarcoma	India	India	*E. coli*/2172	NDM-5
**CP72**	14 years/M	Cardiac arrhythmia with pacemaker implanted	Turkey	Turkey	*K. pneumoniae*/2731	OXA-48
**CP45**	14 years/F	Burkitt Lymphoma	India	India	*E. coli*/648	NDM-5
**CP46**	4 months/F	CHD & RLRTI	Somalia	Somalia	*E. coli*/58	OXA-48
**CP51**	5 years/M	ESRD, HD & Bronchiectasis	Pakistan	Pakistan	*E. coli*/156	OXA-181 & NDM-5
**CP55**[d]	2 months	CHD & NICU admission in Egypt	Egypt	Egypt	*E. coli*/361	OXA-244 &NDM-5
**CP56**[d]	2 months	CHD & NICU admission in Egypt	Egypt	Egypt	*K. pneumoniae*/340	OXA-48 & NDM-1
**CP58**	11 months/M	CP, GDD & NICU	Egypt	Egypt	*K. pneumoniae*/73	NDM-1
**CP59**[e]	2 years/M	CHD & Cardiac surgery	Sudan	India	*K. pneumoniae*/73	NDM-1

**CP60[e]**	2 years/M	CHD & Cardiac surgery	Sudan	India	*K. pneumoniae*/17	NDM-1
**CP63[f]**	1 year/F	Tufting enteropathy	Kuwait	Kuwait	K. oxytoca/135	OXA-48
**CP64[g]**	6 months/F	CP, GDD & RLRT	Egypt	Egypt	*E. coli*/ND	NDM-5
**CP65[g]**	6 months/F	CP, GDD & RLRT	Egypt	Egypt	*K. pneumoniae*/397	NDM-1
**CP85[h]**	6 years/M	Ewing sarcoma	Irak	Irak	*E. coli*/410	NDM-5
**CP86[h]**	6 years/M	Ewing sarcoma	Irak	Irak	*K. pneumoniae*/147	OXA-232 & NDM-5
**CP77**	13 years/F	ALL	Turkey	NO	E. coli/1421	OXA-181
**CP78[i]**	9 months/M	CHD & Epilepsy	Egypt	Egypt	*K. pneumoniae*/45	OXA-48
**CP79[i]**	9 months/M	CHD & Epilepsy	Egypt	Egypt	*Citrobacter* spp./ND	OXA-48
**CP80**	8 years/M	CP, hydrocephalus & VPS	India	India	*E. coli*/410	OXA-181
**CP82**	2 years/M	Congenital hyperinsulinism	Egypt	Egypt	*K. pneumoniae*/39	NDM-1
**CP84**	23 months/	Chromosomal abnormality, CHD, GDD	India	NO	*E. coli*/10	OXA-181
**CP85N[j]**	11 years/F	Medulloblastoma, tumor excision, VPS insertion	India	India	*K. pneumoniae*/16	OXA-181 & NDM-5
**CP87**	6 years/M	Mitochondrial disease	India	India	*E. coli*/410	OXA-181
**CP89[k]**	3 months/M	CHD & Cardiac surgery	Egypt	Egypt	*K. pneumoniae*/70	NDM-5

**CP90**[k]	3 months/M	CHD & Cardiac surgery	Egypt	Egypt	*E. coli*/405	NDM-5
**CP96**[l]	17 years/M	Aplastic anemia	Egypt	NO	*E. coli*/410	OXA-244

CP: Cerebral palsy; GDD: Global developmental delay; CHD: Congenital heart disease; RLRTI: Recurrent lower respiratory tract infections; HLH: Hemophagocytic lymphohistiocytosis; VPS: Ventriculoperitoneal shunt; NICU: Neonatal Intensive Care Unit; ESRD: End-stage renal disease; HD: Hemodialysis; AAL: Acute lymphoblastic leukemia.

[a]Detected during a weekly screening in the second week of hospitalization in the Pediatric Intensive Care Unit.

[b]Detected simultaneously on the same patient shortly after returning from a holiday trip in India where the patient required healthcare. This patient developed two bloodstream infections 7 and 8 months later caused by different *E. coli* and *K. pneumoniae* species producing the same carbapenemase types detected during the first positive admission screening.

[c]Detected on the same patients during two different admission screenings.

[d, e, g, h]Detected on the same patients simultaneously during admission screenings.

[f]Detected during a weekly screening after 2 weeks of hospitalization in a General Pediatric Unit.

[i]Detected simultaneously on the same patient during a weekly screening in the second week of hospitalization in the Pediatric Intensive Care Unit.

[j]Developed a bloodstream infection caused by the same species producing the same carbapenemase 2 weeks later.

[k]Detected simultaneously on the same patient during a weekly screening on hospital day 20 in a General Pediatric Unit.

[l]Developed a bloodstream infection caused by the same species producing the same carbapenemase 3 weeks later.

Supplementary Table S2. Carbapenemen-producing Enterobacterales isolated from fecal carriers without foreign travel history.

ID	Age/gender	Underlying condition	Previous hospital admissions	Species/ ST	Enzyme
**CP1[a]**	4 years/M	Waardenburg syndrome & Hirschsprung disease	YES	*K. pneumoniae*/ 429	NDM-1
**CP4[a]**	4 years/M	Waardenburg syndrome & Hirschsprung disease	YES	*E. coli*/127	NDM-1
**CP11**	6 months/F	Down syndrome, CHD	YES	*E. coli*/2206	OXA244
**CP26**	2 years/M	Chiari malformation & Myelomeningocele & VPS	YES	*E. coli*/410	NDM-5
**E431**	3 months/M	Previously healthy, Circumcision	YES	*E. coli*/38	OXA-244
**CP30**	2 years/F	ALL, Liver abscess & Abdominal surgery	YES	*E. coli*/38	OXA-244
**CP29**	14 years/M	White matter disease, CP & epilepsy	YES	*E. coli*/448	OXA-181
**CP31[b]**	2 years/F	Lysosomal storage disease, GDD & RLRTI	YES	*E. coli*/10	OXA-181
**CP33**	14 years/F	Type-1 diabetes	YES	*E. coli*/38	OXA-244
**CP34[c]**	4 years/F	CP, GDD, Hydrocephalus & VP shunt	YES	*E. coli*/38	OXA-244
**CP38**	11 months/F	GDD, RLRTI Hirschsprung disease	YES	*E. coli*/38	OXA-48
**CP39**	14 months/F	CHARGE syndrome & CHD	YES	*E. coli*/13	NDM-1
**CP40**	16 years/F	CP, GDD & Orthopedic surgeries	YES	*E. coli*/38	OXA-244

**CP41**	1 year/M	Cystic fibrosis & Pancreatic insufficiency	YES	*K. pneumoniae*/ 73	NDM-5
**CP42[b]**	2 years/F	Lysosomal storage disease, GDD & RAP	YES	*E. coli*/450	OXA-181
**CP43[b]**	2 years/F	Lysosomal storage disease, GDD & RAP	YES	*K. pneumoniae*/ 4961	OXA-181
**CP57**	11 years/M	CP, epilepsy & Orthopedic surgeries	YES	*E. coli*/354	OXA-181
**CP61**	10 days/F	Neonatal apnea & NICU stay	YES	*E. coli*/167	NDM-5
**CP66**	14 years/M	Liver transplant	YES	*E. coli*/410	OXA-181
**CP75[d]**	9 years/M	Previously healthy	NO	*E. coli*/38	OXA-244
**CP86N[e]**	17 years/M	Hodgkin's disease	YES	*E. coli*/167	OXA-244
**CP81**	2 years/M	Mucopolysaccharidosis	YES	*E. coli*/617	NDM-5
**CP93**	3-years/M	CML	YES	*E. coli*/405	NDM-5
**CP94**	13 days/M	GAS sepsis, NICU stay	YES	*K. pneumoniae*/17	NDM-7
**CP95**	7 months/F	Previously healthy	NO	*E. coli*/648	NDM-7
**CP98**	9 years/M	Brain tumor, tumor excision	YES	*E. coli*/38	OXA-244
**CP99**	2 years/M	CP, GDD & NICU	YES	*K. pneumoniae*/14	OXA-48 & NDM-1
**CP101**	6 years/F	Previously healthy	NO	*E. coli*/38	OXA-244
**CP102**	3 years/M	Primary immunodeficiency	YES	*K. pneumoniae*/ND	NDM-5

CHD: Congenital heart disease; VPS: Ventriculoperitoneal shunt; AAL: Acute lymphoblastic leukemia; CP: Cerebral palsy; GDD: Global developmental delay; RLRTI: Recurrent lower respiratory tract infections; RAP: Recurrent aspiration pneumonia; NICU: Neonatal Intensive care Unit; CML: Chronic myeloid leukemia; GAS: Group A *Streptococcus*; ND: Not determined.

[a]Detected on the same patient simultaneously during admission screening.

[b]Detected on the same patient. CP31 during the first admission screening, whereas CP42 and CP43 were detected simultaneously during a new admission screening 3 months later.

[c]Detected during a weekly screening after 4 weeks in a General Pediatric Unit.

[d]Detected during a weekly screening in the second week of hospitalization in the Pediatric Intensive Care Unit.

[e]Detected during a weekly screening after 2 weeks of hospitalization in a General Pediatric Unit.

	2018	2019	2020	Quarterly average pre-travel restrictions	Quarterly average post-travel restrictions
**Pediatric admissions**	11,760	20,646	20,583	4575	5165
**PICU admissions**	838	1356	1064	303	253
**NICU admissions**	541	725	794	177	197
**Rectal swabs and positivity rate (%)**	3013 (0.9%)	6555 (0.37%)	6778 (0.19%)	1390 (0.54)	1660 (0.08)

Supplementary Table S3. First three columns show the annual number of Pediatric, PICU, and NICU admissions during the study period. Also, the annual number of rectal screening swabs and the corresponding positivity rate are shown in these columns. Last two columns show quarterly average of admissions, rectal swabs, and positivity rate before (second quarter 2018 to first quarter 2020) and after (second quarter 2020 to fourth quarter 2020) the implementation of COVID-19 related travel restrictions in Qatar.

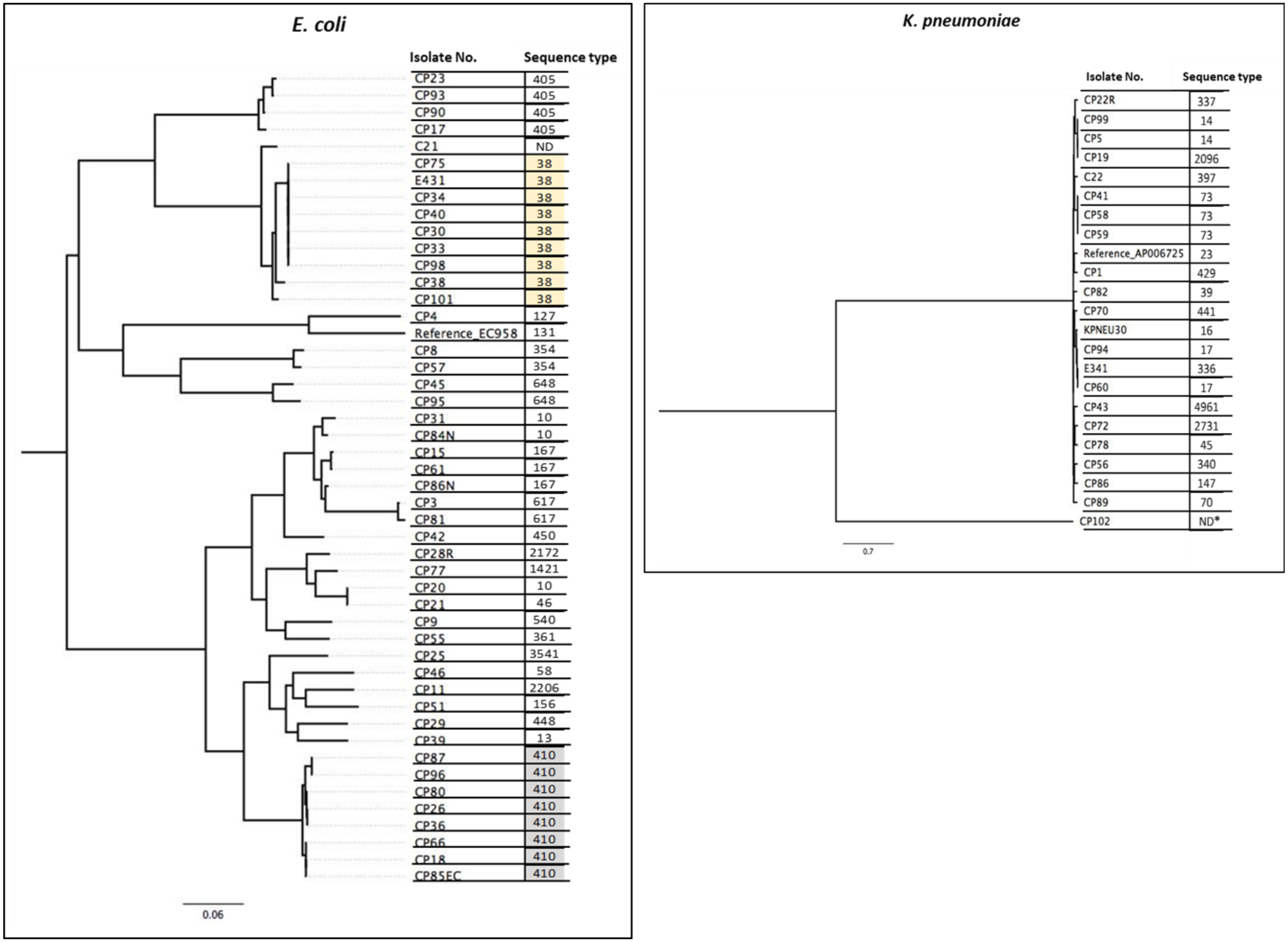

Supplementary Figure S1. Dendograms showing genetic relatedness of 47 carbapenemase-producing *Escherichia coli* and 22 *Klebsiella pneumonia* isolates. **K. quasipneumoniae* subsp *quasipneumoniae*
